# Construction of mouse cochlin mutants with different GAG-binding specificities and their use for immunohistochemistry

**DOI:** 10.1042/BCJ20220339

**Published:** 2023-01-06

**Authors:** Karin Murakami, Ryo Tamura, Sanae Ikehara, Hayato Ota, Tomomi Ichimiya, Naoki Matsumoto, Hisahiro Matsubara, Shoko Nishihara, Yuzuru Ikehara, Kazuo Yamamoto

**Affiliations:** 1 Department of Integrated Biosciences, Graduate School of Frontier Sciences, The University of Tokyo, Kashiwa, Chiba, Japan; 2 Graduate School of Medicine, Chiba University, Chiba, Chiba, Japan; 3 Department of Bioinformatics, Graduate School of Engineering, Soka University, Hachioji, Tokyo, Japan; 4 Glycan and Life System Integration Center (GaLSIC), Soka University, Hachioji, Tokyo, Japan

**Keywords:** biotechnology, glycobiology, glycosaminoglycans, pathology

## Abstract

Glycosaminoglycan (GAG) is a polysaccharide present on the cell surface as an extracellular matrix component, and is composed of repeating disaccharide units consisting of an amino sugar and uronic acid except in the case of the keratan sulfate. Sulfated GAGs, such as heparan sulfate, heparin, and chondroitin sulfate mediate signal transduction of growth factors, and their functions vary with the type and degree of sulfated modification. We have previously identified human and mouse cochlins as proteins that bind to sulfated GAGs. Here, we prepared a recombinant cochlin fused to human IgG-Fc or Protein A at the C-terminus as a detection and purification tag and investigated the ligand specificity of cochlin. We found that cochlin can be used as a specific probe for highly sulfated heparan sulfate and chondroitin sulfate E. We then used mutant analysis to identify the mechanism by which cochlin recognizes GAGs and developed a GAG detection system using cochlin. Interestingly, a mutant lacking the vWA2 domain bound to various types of GAGs. The N-terminal amino acid residues of cochlin contributed to its binding to heparin. Pathological specimens from human myocarditis patients were stained with a cochlin-Fc mutant. The results showed that both tryptase-positive and tryptase-negative mast cells were stained with this mutant. The identification of detailed modification patterns of GAGs is an important method to elucidate the molecular mechanisms of various diseases. The method developed for evaluating the expression of highly sulfated GAGs will help understand the biological and pathological importance of sulfated GAGs in the future.

## Introduction

GAGs are single-chain acidic polysaccharides attached to proteoglycans that play essential roles in biological processes, such as cell adhesion, growth, motility, and differentiation. GAGs are located on the extracellular matrix (ECM) and cell surfaces and act as co-receptors that regulate local retention and stabilization of GAG-binding proteins such as growth factors, cytokines, chemokines, and ECM proteins [1]. GAGs are composed of repeating disaccharide units and there are four classes of GAGs based on the component sugars: hyaluronic acid (HA), chondroitin sulfate (CS)/dermatan sulfate (DS), heparan sulfate (HS)/heparin, and keratan sulfate (KS). These disaccharides are modified at multiple positions via sulfation, acetylation, and/or epimerization except in the case of the HA, where sulfation and epimerization do not occur. These enzymatic modifications result in tremendous structural heterogeneity in GAGs, which deeply related to their biological function [2]. As a consequence of their specific binding to several growth factors and morphogens, sulfated GAGs regulate cell differentiation and are involved in epithelial-mesenchymal transition and carcinogenesis [3–5]. In addition, integrins collaborate with sulfated GAGs in the ECM for cell–ECM interactions in cell adhesion and migration [6,7]. Therefore, sulfated GAGs are essential regulators of cancer progression through the modulation of cell differentiation, invasion, and metastasis [8]. HS is composed of repeating disaccharides made of N-acetylglucosamine (GlcNAc) and glucuronic acid (GlcA), which exhibit heterogeneity in the position and number of sulfations. A highly sulfated HS is called heparin, which contains a high ratio of N-sulfated glucosamine (GlcN) instead of N-acetylated GlcN and a high ratio of iduronic acid (IdoA) instead of GlcA. CS is composed of repeating disaccharides made of N-acetylgalactosamine (GalNAc) and GlcA. DS, also known as chondroitin sulfate B (CSB), is a subtype of CS that contains IdoA, which is generated by epimerization at C-5 of GlcA. CS/DS also showed heterogeneity in the position and number of sulfations per disaccharide unit. CS has five major disaccharide structures called O, A, C, D, and E-units (O-unit: GlcA-GalNAc, A-unit: GlcA-GalNAc (4S), B-unit: IdoA (2S)-GalNAc (4S), C-unit: GlcA-GalNAc (6S), D-unit: GlcA (2S)-GalNAc (6S), and E-unit: GlcA-GalNAc (4S, 6S)). The CSs that contain a high ratio of A, C, D, and E units, are called CSA, CSC, CSD, and CSE, respectively [9]. Such heterogeneity in GAGs makes it difficult to determine the relationship between the structure and function of GAGs.

Several studies have shown the functional importance of GAGs, particularly in the progression of cancer [10]. However, regulation of their expressions and their roles are largely unknown. It is difficult to predict which GAGs are expressed in the cell from the gene expression pattern as hundreds of proteins (such as enzymes involved in GAG biosynthesis and core proteins of proteoglycans) are involved in GAG expression. Moreover, methods for the preparation and recognition of GAGs to determine their fine structures are limited. These limitations make it difficult to study the function of each GAG. For example, mass spectrometry (MS)-based approaches have been established for the analysis of GAGs [11], and endo-type glycosidases such as heparanase I, II, III, and endo-type chondroitinase ABC [12] are simple methods for the digestion or removal of GAGs from the cell surface [13]. A few approaches do exist for visualizing GAGs. Various cationic dyes such as Alcian Blue, Toluidine Blue, and Amido Black have been widely used for the detection and visualization of GAGs in biological samples. However, such dyes have a number of drawbacks, including poor biocompatibility, which is not suitable for intravital staining, and poor selectivity, which usually causes a high background and low sensitivity [14]. Several probes have been reported that can detect GAGs, including anti-HS/heparin antibody (10E4 available from ASMBIO), anti-CSE antibody [15], a branched peptide NT4 that binds to HS/heparin and CS [8], and the malarial protein VAR2CSA, which binds to CSA [16]. There are also considerable studies on GAG-binding scFv antibody using phage display system [17] and FGF family with GAG affinity [18]. Thus, there is a number of reagents, each has a different level of selectivity for classes of GAG structures, but no reagent recognizes a unique structure and consequently, given the diversity of structure in HS, new reagents are of substantial interest. Recently, we identified cochlin as a GAG-binding lectin [19]. Cochlin is a major component of the cochlear extracellular matrix. The gene *COCH*, which codes for cochlin, is responsible for late-onset non-syndromic autosomal dominant hearing loss 9 (DFNA9) [20,21]. Cochlin is a secreted protein composed of three domains: the N-terminal LCCL (Limulus factor C, cochlin, and late gestation lung protein Lgl1) domain and two von Willebrand factor A (vWA)-like domains (vWA1 and vWA2). Most mutations found in the DFNA9 patients are located in the LCCL domain. However, the molecular mechanisms by which cochlin is related to hearing remain largely unknown [22,23]. We focused on cochlin as the starting point of the development of GAG-recognition probes by preparing several domain-deleted and amino acid-substituted cochlin fused to human IgG-Fc.

In this study, we determined the GAG-binding specificity of cochlin and its mutants using various binding assays. We found that some of the cochlin mutants could be used as specific probes against heparin and CSE, both of which are highly sulfated GAGs. Furthermore, we demonstrated that cochlin is applicable in flow cytometry, histochemical staining, pull-down assays, and lectin blotting. This probe can be applied for early detection of cancer and establishment of new diagnostic methods in the future.

## Results

### Cochlin binds to highly sulfated GAGs

A previous study by our group showed that human and mouse cochlins bind to heparin [19]. To further investigate the glycan-binding specificity of cochlin, evanescent-field fluorescence detection was conducted using recombinant wild-type mouse cochlin (mCOCH(FL))-Fc (mouse cochlin and amino acid residues 27–552 were fused to the myc-tag at the N-terminus and human IgG-Fc region at the C-terminus). The glycan microarray for evanescent-field fluorescence detection was used to investigate the sugar-binding specificity of mCOCH(FL)-Fc, and contained 16 monosaccharides and 59 oligosaccharides including six GAGs (HA, CSA, DS, HS, heparin, and KS), and 21 glycoproteins [24]. Mock-Fc (myc-tag fused to human IgG-Fc) did not bind to any saccharide. In contrast, mCOCH(FL)-Fc specifically bound to heparin and did not bind to any other spots (Supplementary Figure S1). Thus, mCOCH (FL)-Fc is expected to be a good candidate for a heparin detection probe.

**Figure 1.**
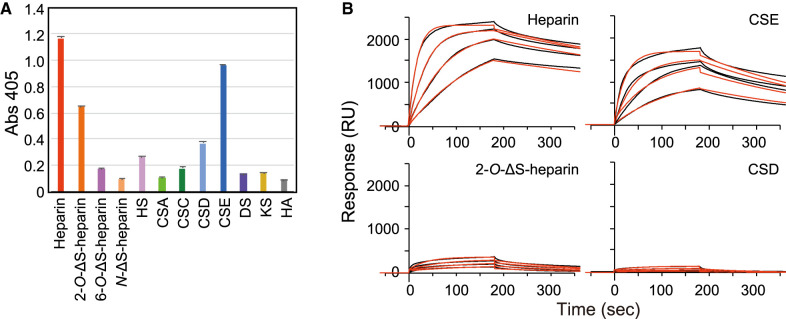
Mouse cochlin (full length; residues 27–552) fused to Fc binds to highly sulfated GAGs. (**A**) Cochlin-Fc binding was examined using indirect ELISA. Biotinylated GAGs were immobilized on 96-well streptavidin-coated microtiter plates. Cochlin-Fc was added to the wells at the concentration of 1.5 µg/ml. The absorbances at 405 nm represent the mean ± standard deviations (*n* = 3). 2-*O*-desulfated heparin (2-*O*-ΔS heparin), 6-*O*-desulfated heparin (6-*O*-ΔS heparin), *N*-desulfated heparin (*N*-ΔS heparin), HS: heparan sulfate; CSA: condroitin sulfate A; CSC: condroitin sulfate C; CSD: condroitin sulfate D; CSE: condroitin sulfate E; DS: dermatan sulfate; KS: keratan sulfate; HA: hyaluronic acid. (**B**) Surface plasmon resonance sensorgram showing the binding kinetics of cochlin-Fc to the immobilized GAGs; heparin, CSE, 2-*O*-ΔS-heparin and CSD. Data for each concentration (1.25 µg/ml, 2.5 µg/ml, 5 µg/ml, or 10 µg/ml) of cochlin mutants are shown as black lines, and the calculated fit with a 1 : 1 binding model is shown as red lines.

Next, to investigate the binding ability of cochlin to GAGs in detail, an ELISA was conducted using recombinant mCOCH(FL)-Fc (Figure 1A). Twelve types of biotinylated GAGs, containing six types of GAGs that were not contained in the previous glycan microarray (2-*O*-desulfated (ΔS)-heparin, 6-*O*-ΔS-heparin, *N*-ΔS-heparin, CSC, CSD, and CSE) were immobilized on the plate. The results showed that mCOCH(FL)-Fc binds not only to heparin but also to CSE, both of which are highly sulfated subtypes of GAGs (Figure 1A). To quantitatively compare the binding ability of cochlin to GAGs, the binding kinetics of mCOCH(FL)-Fc to biotinylated GAGs was calculated by BIAcore (surface plasmon resonance (SPR) detector) using recombinant mCOCH(FL)-Fc (Figure 1B, Table 1). The *K_D_* values of mCOCH(FL)-Fc to heparin, CSE, 2-*O*-ΔS-heparin, and CSD were 1.8 × 10^−9^, 1.4 × 10^−8^, 4.4 × 10^−8^, and 1.9 × 10^−7^ M, respectively. Comparing ELISA data (including inhibition assay, Supplementary Figure S2) and SPR data on various GAG-binding specificities of Cochlin-Fc protein, the order of highest binding was consistent with heparin, CSE, 2-O-desulfated heparin, and CSD. However, the signal ratio was different between the data obtained from ELISA and SPR (and aslo from inhibition assay using ELISA). The reason for this discrepancy may be the effect of biotinylation method in each system and the difference in immobilization efficiency.

**Table 1:** Kinetic parameters for the interaction of cochlin mutants with immobilized GAGs

Protein^1^	Expression region	GAG	*k_a_* (M^−1^s^−1^)	*k_d_* (s^−1^)	*K*_D_ (nM)	*R*_max_ (RU)
FL	27–552	Heparin	(6.70 ± 0.05) × 10^5^	(1.19 ± 0.01) × 10^−3^	1.78	2204 ± 3
FL	27–552	CSE	(2.00 ± 0.01) × 10^5^	(2.77 ± 0.03) × 10^−3^	13.9	1650 ± 6
FL	27–552	2-*O*-ΔS-heparin	(1.11 ± 0.01) × 10^5^	(4.86 ± 0.05) × 10^−3^	43.8	342 ± 2
FL	27–552	CSD	(5.12 ± 0.31) × 10^4^	(9.63 ± 0.52) × 10^−3^	188	167 ± 3
ΔLCCL_a	130–552	Heparin	(3.13 ± 0.05) × 10^5^	(2.23 ± 0.03) × 10^−3^	7.16	1738 ± 5
ΔLCCL_a	130–552	CSE	(9.60 ± 0.08) × 10^4^	(1.94 ± 0.04) × 10^−3^	20.2﻿	1544 ± 8
ΔLCCL_a	130–552	2-*O*-ΔS-heparin	(8.35 ± 0.14) × 10^4^	(6.74 ± 0.09) × 10^−3^	80.7	91 ± 1
ΔLCCL_a	130–552	CSD	(7.55 ± 0.25) × 10^4^	(2.14 ± 0.07) × 10^−2^	284	175 ± 2
ΔvWA2	27–365	Heparin	(1.72 ± 0.05) × 10^5^	(7.75 ± 0.14) × 10^−4^	4.50	2206 ± 4
ΔvWA2	27–365	CSE	(8.72 ± 0.02) × 10^4^	(7.07 ± 0.10) × 10^−4^	8.11	2487 ± 4
ΔvWA2	27–365	2-*O*-ΔS-heparin	(2.13 ± 0.04) × 10^4^	(2.27 ± 0.04) × 10^−3^	107	1714 ± 21
ΔvWA2	27–365	CSD	(8.11 ± 0.02) × 10^3^	(2.84 ± 0.05) × 10^−3^	351	2357 ± 47

1 These proteins were fused to human IgG-Fc.

**Figure 2.**
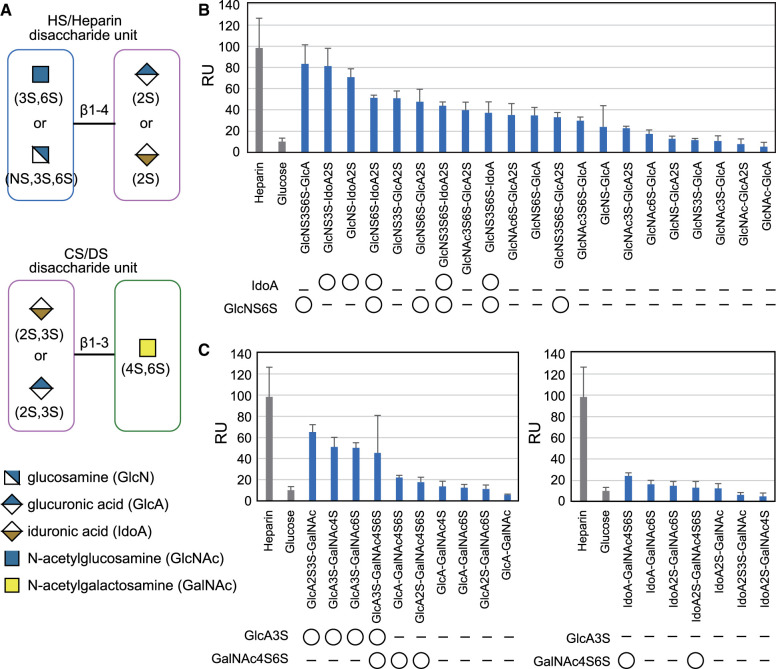
Binding of cochlin-ProA to chemically synthesized disaccharide units of HS/heparin and CS/DS analyzed by surface plasmon resonance. (**A**) Schematic illustration of the disaccharide units of HS/heparin and of CS/DS. (**B** and **C**) mCOCH(FL)-ProA expressed by S2 cells were used at the concentration of 0.4 µM. Cochlin-binding with several disaccharide units from HS and heparin (**B**) and with several disaccharide units from CS and DS (**C**). IdoA and GlcNS6S were commonly found in strongly binding disaccharides in B and GlcA3S and GalNAc4S6S residues were commonly found in strongly binding disaccharide in C.

Furthermore, to determine the structural features of the glycan ligand recognized by cochlin, GAG array analysis was conducted using recombinant wild-type mouse cochlin-Protein A (mouse cochlin fused to the protein A-tag at the C-terminus (mCOCH(FL)-ProA)). The GAG array chip was immobilized with heparin as a positive control, glucose as a negative control, and chemically synthesized disaccharide units of GAGs (21 disaccharides of HS/heparin and 17 disaccharides of CS/DS, Figure 2A) [25]. The mCOCH(FL)-ProA strongly bound to the disaccharides containing IdoA or GlcNS3S6S (Figure 2B) in case of heparan sulfate/heparin. However, mCOCH(FL)-ProA did not bind to the units of DS (IdoA-GalNAc), which also contained IdoA (Figure 2C), suggesting that cochlin binds to heparin by recognizing IdoA adjacent to GlcN. In contrast, mCOCH(FL)-ProA strongly bound to the disaccharides containing GlcA3S (K-unit), which is a characteristic of CSK and also found in CSE [26] (Figure 2C), suggesting that cochlin could bind to CSE by recognizing the GlcA3S structure. Moreover, cochlin seemed to have a strong binding to GAGs whose amino sugar residues are sulfated at two sites especially at NH_2_ and 6-OH groups in both heparan sulfate and chondroitin sulfate glycosaminoglycans.

### Truncation of domains of cochlin alters its GAG-binding specificity

**Figure 3.**
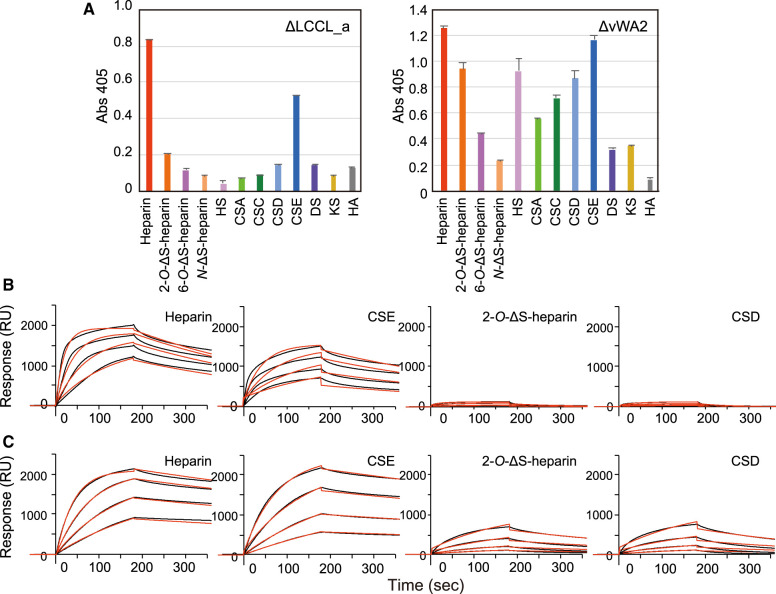
GAG-binding specificities of cochlin-Fc mutants (ΔLCCL_a; residues 130–552 and ΔvWA2; residues 27–365). (**A**) The binding activity of each cochlin mutant fused to human IgG-Fc was examined using indirect ELISA. Biotinylated GAGs were immobilized on streptavidin-coated microtiter plates and cochlin mutants were added to the wells at the concentration of 1.5 µg/ml. The values represent the mean ± standard deviations (*n* = 3). (**B** and **C**) Surface plasmon resonance sensorgram showing the binding kinetics of cochlin mutants, ΔLCCL_a (**B**) and ΔvWA2 (**C**), to immobilized heparin, CSE, 2-*O*-ΔS-heparin and CSD, respectively. Raw data for each concentration (1.25 µg/ml, 2.5 µg/ml, 5 µg/ml, or 10 µg/ml) of cochlin mutants are shown as black lines, and the calculated fit with a 1 : 1 binding model is shown as red lines.

To determine the residues of mouse cochlin involved in sugar recognition, two truncated mutants, one lacking an N-terminal LCCL domain (ΔLCCL_a; residues 130–552) and the other lacking the C-terminal vWA2 domain (ΔvWA2; residues 27–365) were prepared (Supplementary Figure S3). First, the binding specificities of the mutants were investigated using ELISA. ΔLCCL_a fused to Fc (ΔLCCL_a-Fc) and showed almost the same binding to highly sulfated GAGs, heparin, and CSE as mCOCH(FL)-Fc and less binding to 2-*O*-ΔS-heparin, HS, and CSD than mCOCH(FL)-Fc, indicating that ΔLCCL_a-Fc is more specific to highly sulfated GAGs (Figure 3A) than mCOCH(FL)-Fc. On the other hand, the GAG-binding specificity for ΔvWA2-Fc was dramatically changed to broad specificity against various sulfated GAGs (Figure 3A). The cochlin mutant, which is only composed of an LCCL domain (residues 27–130, 27–153 and 27–158) and the mutants that lacked both LCCL and vWA1 domains (residues 355–552) were prepared and tested for their ability to bind GAGs, and neither of them could bind to GAGs (Supplementary Figures S3, S4). These results suggest that the vWA1 domain is essential for binding to GAGs and that LCCL and vWA2 domains are also involved in the interaction with GAGs. The LCCL domain itself may not have the ability to bind GAGs but may enhance the binding ability of vWA domains for several GAGs, and the vWA2 domain restricts the binding specificity of cochlin to heparin and CSE.

The binding kinetics of wild-type cochlin (FL) (Figure 1B) and its mutants, including ΔLCCL_a (Figure 3B) and ΔvWA2 (Figure 3C), to GAGs was calculated by SPR using purified recombinant proteins. The *K_D_* values of ΔLCCL_a to heparin, CSE, 2-*O*-ΔS-heparin, and CSD were 7.9 × 10^−8^, 1.2 × 10^−8^, 4.0 × 10^−7^, and 4.5 × 10^−7^ M, respectively (Table 1). The *K_D_* of ΔvWA2 to heparin, CSE, 2-*O*-ΔS-heparin and CSD were 1.5 × 10^−8^, 4.6 × 10^−8^, 3.0 × 10^−7^ and 6.0 × 10^−7^ M, respectively. Interestingly, ΔvWA2 showed higher Rmax values for 2-*O*-ΔS-heparin and CSD than for FL and ΔLCCL_a (Table 1). It is generally known that Rmax does not vary with the analyte. However, in this experiment, the apparent amount of ligand on the GAG molecule increased due to the difference in ligand specificity of the probes. Therefore, it is suggested that ΔvWA2 binds to both 2-*O*-ΔS-heparin and CSD because the lack of vWA2 domain decreases the target specificity and allows the protein to bind to various types of microstructures on GAG.

### N-terminal amino acid residues of cochlin contribute its specific binding to heparin

**Figure 4.**
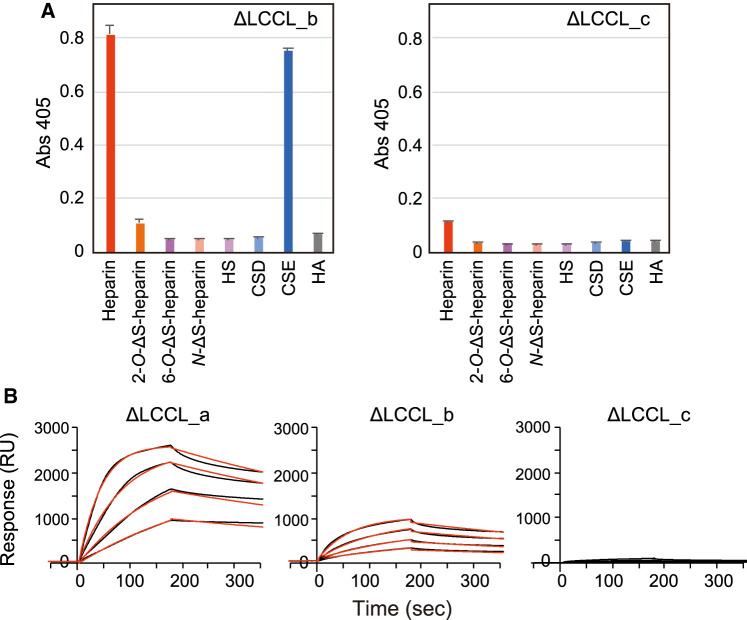
GAG-binding specificities of cochlin-Fc mutants (ΔLCCL_a; residues 130–552, ΔLCCL_b; residues 153–552 and ΔLCCL_c; residues 158–552). (**A**) The binding activity of each cochlin mutant was examined by indirect ELISA. Biotinylated GAGs were immobilized on 96-well streptavidin-coated microtiter plates. Cochlin-Fc mutants were added to the wells at the concentration of 1.5 µg/ml. The values represent the mean ± standard deviations (*n* = 2). (**B**) Surface plasmon resonance sensorgram showing the binding kinetics of cochlin-Fc mutants, ΔLCCL_a, ΔLCCL_b, and ΔLCCL_c to immobilized heparin. Raw data for each concentration (1.25 µg/ml, 2.5 µg/ml, 5 µg/ml, or 10 µg/ml) of cochlin mutants are shown as black lines, and the calculated fit with a 1 : 1 binding model is shown as red lines.

Based on the results described above, we hypothesized that residues on the N-terminus of cochlin could play important roles in the binding of cochlin to GAGs. Therefore, we prepared other kinds of cochlin-Fc mutants with truncation at the N-terminus (ΔLCCL_b; residues 153–552 and ΔLCCL_c; residues 158–552) and investigated the binding specificity and binding kinetics of these mutants to heparin using both ELISA and SPR. The results of ELISA showed that ΔLCCL_b-Fc had the same binding specificity as ΔLCCL_a-Fc. In contrast, ΔLCCL_c-Fc was deficient in its GAG-binding ability (Figure 4A). Consistently, SPR results showed that the affinity of ΔLCCL_c-Fc was substantially lower than that of ΔLCCL_a-Fc or ΔLCCL_b-Fc (Figure 4B and Table 2). These results suggest that the part of cochlin from the N-terminus to residue 157, which contains the LCCL domain and the loop region between LCCL and vWA1, plays a key role in enhancing the interaction of cochlin with heparin. Interestingly dibasic KK sequences are found in loop region (from K149 to N161), which could be involved in the binding ability and specificity of GAG binding.

**Table 2:** Kinetic parameters for the interaction of cochlin mutants with immobilized heparin

Protein^1^	Expression region	GAG	*k_a_* (M^−1^s^−1^)	*k_d_* (s^−1^)	*K*_D_ (nM)	*R*_max_ (RU)
FL	27–552	Heparin	(3.74 ± 0.03) × 10^5^	(5.79 ± 0.32) × 10^−4^	1.55	5011 ± 25
ΔLCCL_a	130–552	Heparin	(1.85 ± 0.03) × 10^5^	(1.39 ± 0.02) × 10^−3^	7.49	2705 ± 6
ΔLCCL_b	153–552	Heparin	(1.00 ± 0.04) × 10^5^	(1.62 ± 0.02) × 10^−3^	16.2	1014 ± 3
ΔLCCL_c	158–552	Heparin	(1.66 ± 0.04) × 10^4^	(1.60 ± 0.03) × 10^−3^	96.0	591 ± 11

1 These proteins were fused to human IgG-Fc.

### Detection of highly sulfated GAGs on the surface of GAG-modified CHO cells using cochlin mutants

**Figure 5.**
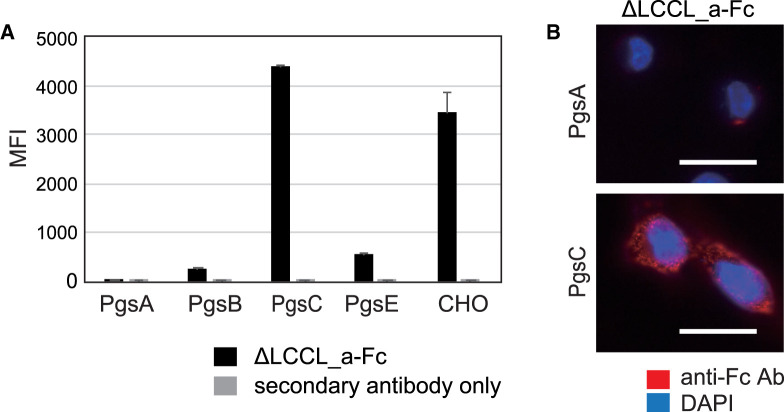
Binding of a cochlin mutant, ΔLCCL_a-Fc, to several GAG-modified cells. (**A**) Relative mean fluorescence intensity (MFI) of CHO cells and its GAG-modified cells stained with recombinant cochlin mutant, ΔLCCL_a-Fc, and the binding was measured by flow cytometry using anti-Fc-PE. The values represent the mean ± standard deviations (*n* = 3). GAG-modified cells are as follows: PgsA (GAG-deficient); PgsB (GAG-deficient); PgsC (normal GAG); PgsE (sulfate deficient for HS/heparin). (**B**) Immunofluorescent images of PgsA and PgsC cells incubated with ΔLCCL_a-Fc and detected by fluorescence microscope using anti-Fc-Dylight550. ΔLCCL_a-Fc staining showed spotted signal for GAG-expressing cells (PgsC) while not for GAG-deficient cells (PgsA). The scale bar represents 20 µm.

As described above, an N-terminal truncation mutant of mouse cochlin, ΔLCCL_a-Fc, showed specific binding to heparin and CSE. Therefore, recombinant ΔLCCL_a-Fc could be used as a probe for detecting highly sulfated GAGs. To evaluate whether ΔLCCL_a-Fc is useful for detecting highly sulfated GAGs on the cell surface, flow cytometry was used to investigate the binding of ΔLCCL_a-Fc to CHO cells and GAG-modified cell lines derived from CHO cells (PgsA, B, C, and E) (Figure 5A). Pgs series of CHO cells are GAG-modified cell lines established by Esko et al. [27,28]. PgsC shows normal GAG-expression compared with CHO cell. In contrast, PgsA and PgsB cells are GAG-deficit cells and PgsE is HS/heparin sulfate-deficient cell. The results showed that cochlin bound to normal GAG-expressing cells (PgsC, CHO) but not to GAG-deficient cells (PgsA and PgsB) or HS/heparin sulfate-deficient cells (PgsE). Additionally, immunofluorescence staining of cell surfaces of PgsA (GAG-deficient cells) and PgsC (GAG-expressing cells) was conducted to confirm the binding specificity of cochlin-Fc to the GAG-modified cells. Visualization was also performed using ΔLCCL_a-Fc. The image showed clear membrane staining, similar to the proteoglycan staining reported previously [8,15,16] for PgsC, but not for PgsA (Figure 5B). These results indicate that ΔLCCL_a-Fc is a useful probe for the detection of highly sulfated GAGs on cell surfaces with a low background.

### Detection of highly sulfated GAGs on the surface of a prostate cancer cell line using a cochlin mutant

**Figure 6.**
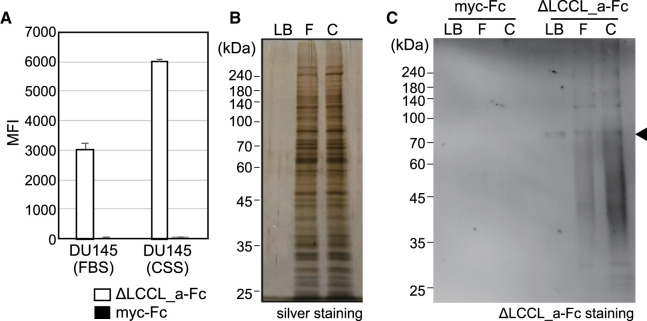
Binding of ΔLCCL_a cochlin mutant to CRPC cells and pull-down assay. (**A**) DU145 cells cultured in the presence of FBS or CSS were incubated with ΔLCCL_a-Fc and its binding was detected by flow cytometry using Phycoerythrin-conjugated anti-Fc Ab. Mean fluorescent intensity (MFI) represents the mean ± standard deviations (*n* = 3). (**B**) The whole-cell lysates from DU145 cells cultured in the presence of FBS or CSS were subjected to SDSD–PAGE and silver staining. (**C**) Whole-cell lysate from DU145 cells cultured in the presence of FBS or CSS were pulled down with Protein G-Sepharose immobilized with myc-Fc (control) or ΔLCCL_a-Fc. The pulled down samples were subjected to SDS–PAGE, blotted onto a PVDF membrane and detected using ΔLCCL_a-Fc and anti-Fc-HRP. LB: Lysis buffer, FBS: whole-cell lysate of DU145 cultured in the presence of FBS, CSS: whole-cell lysate of DU145 cultured in the presence of CSS. The arrow head shows the elution position corresponding to ΔLCCL_a-Fc.

DU145 cells are hormone-resistant human prostate carcinoma cells that have growth ability without androgens, whereas normal prostate cancer cells require androgens for their growth. In the process of castration resistance, we found that androgen receptor-mediated signals were lost in prostate cancer cells cultured under androgen-deprivation conditions, and GAG chain-mediated proliferation signals became dominant instead [29]. The expression levels of highly sulfated GAGs on the surface of DU145 cells cultured in a medium containing 10% FBS or CSS (hormone-deprived serum) were compared by flow cytometry using ΔLCCL_a-Fc (Figure 6A). Interestingly, DU145 cells cultured in the presence of CSS showed a relatively higher signal of cochlin binding than cells cultured in the presence of FBS. In addition, a pull-down assay was conducted using whole-cell lysates of the DU145 cells cultured in a medium containing 10% FBS or CSS and protein G resin immobilized with mock-Fc or ΔLCCL_a-Fc. The results of silver staining of cell lysate derived from two types of cells showed no difference (Figure 6B). In contrast, lectin blotting revealed a smearing pattern only for the proteins pulled down by ΔLCCL_a-Fc and the cells cultured in the presence of CSS was more strogly stained than cells cultured in the presence of FBS (Figure 6C). The band pattern of proteoglycan in electrophoresis shows a smearing pattern because of the heterogeneity of the length of GAGs attached to the proteoglycan core protein. Therefore, proteoglycans attached to highly sulfated GAGs were pulled down by the ΔLCCL_a-Fc-immobilized beads. In addition, consistent with the flow cytometry results, the signal from DU145 cells cultured in the presence of CSS was stronger than that from DU145 cells cultured in the presence of FBS.

### Histochemical detection of GAGs using cochlin-Fc mutant, ΔvWA2-Fc

**Figure 7.**
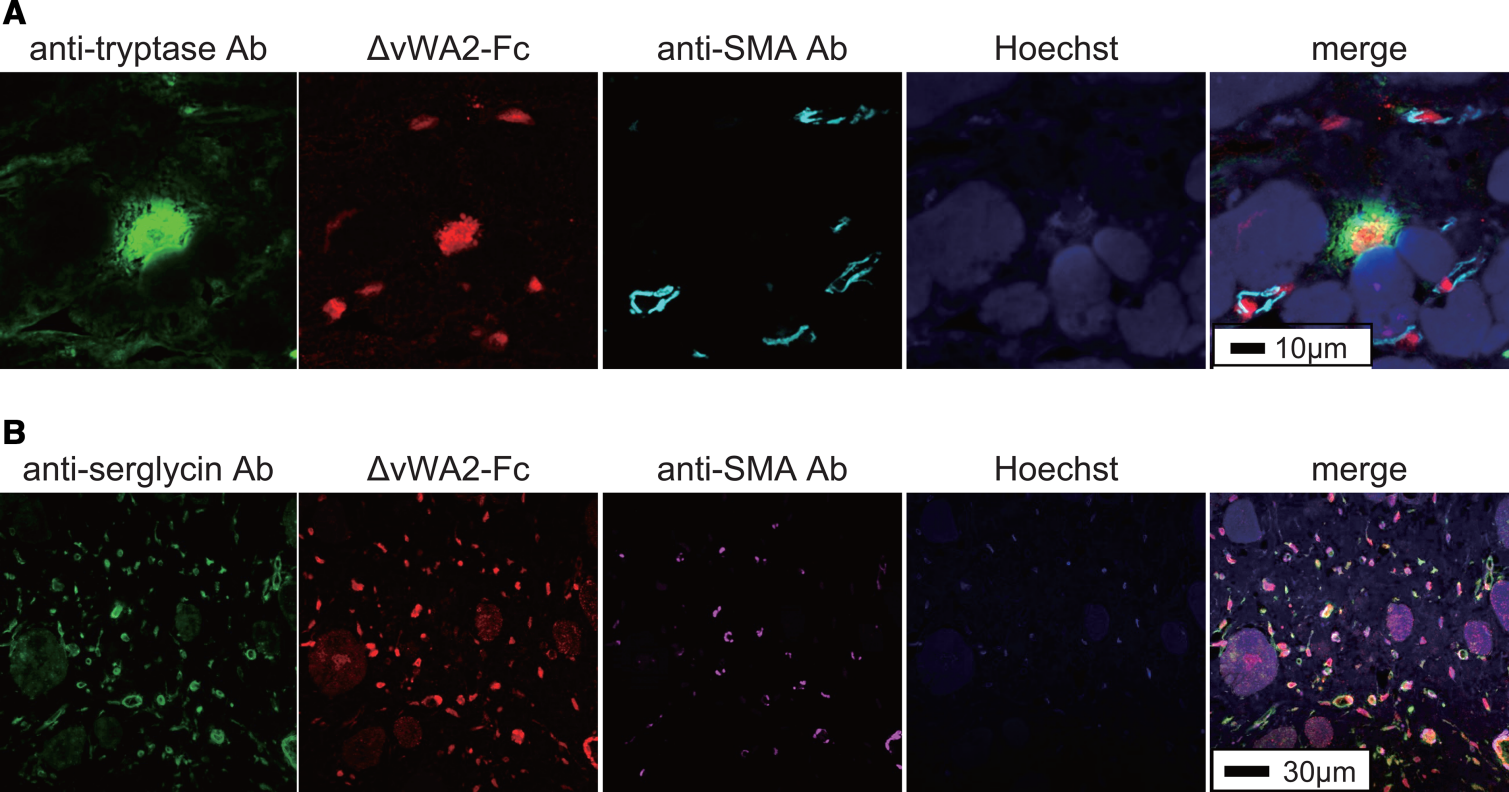
Histochemical staining of pathological specimens from human myocarditis patients. (**A**) Pathological specimens from human myocarditis patients were maltiple stained with anti-tryptase Ab, cochlin mutant, ΔvWA2-Fc, anti-α-smooth muscle actin (SMA) Ab, and Hoechst33342 (Hoechst). Each photo is superimposed in one image (Merge). (**B**) Pathological specimens from human myocarditis patients were maltiple stained with anti-serglycin Ab, cochlin mutant, ΔvWA2-Fc, anti-α-smooth muscle actin (SMA) Ab, and Hoechst33342 (Hoechst). Each photo is superimposed in one image (merge).

To investigate the relationship between disease and the GAG chain, it may be appropriate to first identify the mutants that bind broadly to various types of GAG chains. Therefore, we used the cochlin-Fc mutant ΔvWA2 to stain the pathological specimens. Mast cells are known to increase in the heart during heart failure [30,31]. In addition, histamine or tryptase [32], which act as vasodilators and pro-fibrotic factors secreted by mast cells, as well as renin [33] can promote heart failure. Furthermore, mast cells have multiple subtypes with differential expression of molecules and trigger cell activation. The results of single-cell RNA-seq analysis collected from heart disease tissue also showed CD69 and chemokine expression [34]. It should be noted that the histological identification of mast cells involves toluidine blue staining, which limits histological analysis by multi-fluorescent staining. Pathological specimens from human myocarditis patients were stained with an anti-tryptase antibody and the ΔvWA2-Fc. Mast cell infiltration is often observed in myocardial tissue, and large tryptase-positive mast cells were observed in the tissues of patients with myocarditis (Figure 7A). When these specimens were stained with ΔvWA2-Fc, not only typtase-positive cells but also smaller positive images were observed (Figure 7A). To further understand the tryptase-negative and ΔvWA2-Fc-positive cells, we stained them with anti-serglycin antibody, which is a marker protein of mast cells. As a result, most of the small ΔvWA2-positive images were stained with the anti-serglycin antibody (Figure 7B). Serglycin is well known as a proteoglycan localized in secretory granules in mast cells, and is thought to retain histamine in granules via highly sulfated HS. Taken together, our results show that not only tryptase-positive mast cells but also tryptase-negative mast cells infiltrate the myocardial tissue of myocarditis patients, and a definite diagnosis of myocarditis may be conducted using both conventional anti-tryptase antibodies and the cochlin-Fc mutant. Furthermore, staining with ΔvWA2-Fc may be effective for the early diagnosis of myocarditis.

## Discussion

Several studies have indicated that GAG chains have diversity in sulfation and epimerization in their disaccharide repeating units, and it is important to investigate the differences in such structures because growth factors and chemokines could bind to these specific structures of GAGs [18]. However, the variety of GAGs expressed in the cell is difficult to predict from gene expression patterns as several proteins are involved in GAG expression, which makes it difficult to study the function of each GAG. When we focus on the function of GAGs, it is important to consider that as for the interaction with proteins, although GAGs play the role of a polyanion, the variety of fine structures of GAGs play the role of specific active domains for interactions with particular GAG-binding proteins [35]. Therefore, a probe that can simply detect intact GAGs expressed on the cell surface by distinguishing precise GAG structures should be a powerful tool to enhance the investigation of distinct functions of GAGs.

As the sulfation of GAG is increased in cancer [36], highly sulfated GAGs are potentially available as biomarkers of cancer malignancy. In particular, the detection of heparin or CSE expression on the surface of cancer cells has great significance, as explained below. Several studies have indicated the function of heparin. However, knowledge of its biological roles is limited. Heparin is biosynthesized by mucosal mast cells [37–39] in mammalian tissues, such as the intestine, lung, and liver [40], and heparin prepared from animals has been widely used as a clinical anticoagulant drug [41,42]. However, compared with HS, which is generally expressed by all cells, the distribution of heparin is limited. In addition, heparin has many biological activities, such as anticoagulation, cell proliferation, angiogenesis, and interaction with various proteins, some of which are shared with HS [43]. These studies have suggested possible roles of sulfated HS/heparin but not the specific role of heparin. Several other studies have suggested that heparin is a proteoglycan conjugated with the core protein serglycin expressed in mast cells. However, no other core proteins that are attached to heparin have been reported. On the other hand, CSE is abundantly present in squid cartilage, but mammalian cells rarely express CS containing highly sulfated E-units. However, it is assumed that CS, which partially contains E-units, will be expressed on the surface of cancer cells whose sulfation on GAGs is increased [15]. Consequently, to understand the role of highly sulfated GAGs, heparin, and CSE, it is necessary to detect highly sulfated GAGs and identify the core proteins of these GAGs. Therefore, the analysis of the expression of highly sulfated GAGs on the cell surface conducted in this study is important.

In this study, we demonstrated that mouse cochlin and its mutants are probes that can be used to detect GAGs, but not other glycans expressed on the cell surface, which makes it easy to study the conditions under which the expression of highly sulfated GAGs is increased. Currently, a probe which can clearly distinguish between heparin and HS and between CSE and other CSs is not available. Thus, cochlin is particularly valuable in future GAG research. The results from ELISA and SPR showed that cochlin and its mutant ΔLCCL_a specifically binds to heparin and CSE as strongly as the general antibody binds to antigen, which indicates that it will be useful for detecting heparin or CSE in various experiments, such as visualization of ligand saccharides on cells or pathological specimens, flow cytometry, pull-down assays, and Western blotting. In this study, the use of GAG-modified cells and cancer cells revealed that cochlin is a useful probe for flow cytometry, lectin staining of cells or tissue slices, pull-down assay, and lectin blotting (Figures 5–7). Similar to the visualization of GAGs with previously reported probes [8,15,16], a spotted signal was observed in the cell surface stained with cochlin (Figure 5B). GAGs such as HS, heparin, CS, and DS, expressed as proteoglycans, are considered to form clusters that serve as a scaffold for signal transduction of the GAG-binding proteins. Each spot probably indicated a GAG cluster. In this study, Western blotting of proteins pulled down with cochlin showed a smearing pattern, which suggests it will be possible that proteins contained in highly sulfated GAG clusters could be identified by pull-down assays using cochlin as bait (Figure 6C) and identification of the core protein(s) would be of great help in comprehensively understanding the biology of GAG chains.

Based on the GAG-binding specificities of constructed cochlin mutants, it was suggested that the vWA1 domain was essential for binding of cochlin to GAGs, and that the vWA2 domain and the loop region between the LCCL and vWA1 domains were involved in the interaction, which specifically recognizes highly sulfated GAGs by cochlin in a coordinated manner (Supplementary Figure S5). Interestingly, the GAG-binding specificity broadened when cochlin lacked the vWA2 domain. Initially, we assumed that the lack of a domain may decrease the binding ability against GAGs. However, it broadened the ligand specificity. It is possible that residues located on the cleft between vWA1 and vWA2 domains sandwich GAGs to specifically recognize heparin and CSE. To validate our predicted mechanism of recognition of GAGs by cochlin, the surface charge of cochlin was calculated from the homology model of cochlin, which suggested that the vWA1 domain has a positively charged patch and that several basic amino acids exist in the cleft between the vWA1 and vWA2 domains (Supplementary Figure S6). Additionally, the mutants with truncation at the N-terminus showed significantly reduced GAG-binding ability. Truncation of cochlin from the N-terminus to residue 157 (ΔLCCL_c) significantly reduced the GAG-binding ability (Table 2). It should be noted that the mutants that were only composed of an LCCL domain could not bind to GAGs (Supplementary Figure S4). Therefore, the residues from N-terminal to residue 157 are considered to play an important role in facilitating the binding of the vWA1 domain to GAGs, although the LCCL domain did not bind to GAGs by itself. Notably, each mutant constructed in this study exhibited different GAG-binding specificities and affinities. Using these cochlin mutants in combination to detect GAGs, the GAG modification pattern can be explained in detail.

In conclusion, we established a GAG detection system using recombinant mouse cochlin-Fc and its mutants, one of which specifically reacts with heparin and CSE. These probes are applicable for ELISA, flow cytometry, and immunohistochemical analyses. These might be helpful for the elucidation of the biological and pathological significance of highly sulfated GAGs.

## Methods

### Reagents and cell lines

CHO-K1 (ATCC CCL-61) and mutant CHO cell lines PgsA-745 (ATCC CRL-2242), PgsB-618 (ATCC CRL-2241), PgsC-605 (ATCC CRL-2245), PgsD-677 (ATCC CRL-2244), and PgsE-606 (ATCC CRL-2246) were obtained from the American Type Culture Collection. The prostate cancer cell line DU145 was kindly provided by Professor Shoko Nishihara, Soka University. The cells were cultured in RPMI 1640 medium (Gibco, Tokyo, Japan) supplemented with 10% v/v FBS (Gibco) or CSS (Gibco), 100 U/ml penicillin G, and 100 µg/ml streptomycin. Purified chondroitin sulfate A (CSA) from whale cartilage, chondroitin sulfate C (CSC) from shark cartilage, dermatan sulfate (DS) from porcine skin, keratan sulfate (KS) from porcine nasal cartilage, and heparan sulfate (HS) from porcine kidney were purchased from PG research (NaCS-A2, NACS-C2, NADS-B2, NSKS2, and NaHS-P2, Tokyo, Japan, http://www.pg-r.com). Hyaluronic acid (HA) from *Streptococcus zooepidemicus* was provided by Shiseido Co. (Tokyo, Japan). Chemically modified 2-*O*-ΔS-heparin, 6-*O*-ΔS-heparin, *N*-ΔS-heparin, and purified HS were obtained from Iduron (DSH001/2, DSH002/6, and DSH004/NAc, Manchester, U.K.). Purified chondroitin sulfate D (CSD) from shark cartilage (NaCS-D2(Shc)) and biotinylated heparin from porcine intestine (BHHep-Na(PgI)) were obtained from PG Research. Purified chondroitin sulfate E (CSE) from squid cartilage (034-23061) was obtained from Wako Pure Chemicals (Tokyo, Japan). GAGs except for heparin were biotinylated via carboxyl or aldehyde groups by cross-linking. Reaction solutions [1 mg/ml GAG in 100 mM MES-NaOH (pH 5.3), 1.25 mM EZ-link hydrazide biotin (Thermo Fisher Scientific, Tokyo, Japan) and 125 µg/ml 1-ethyl-3-(3-dimethylaminopropyl)carbodiimide (Thermo Fisher Scientific)] were incubated at 20°C for 12 h in the dark. The solution was dialyzed against PBS.

### Expression and purification of recombinant proteins

The cDNA encoding mouse cochlin (Genbank: NM_00729.5) was amplified by PCR from mouse spleen cDNA, using primers mcochlin-F and mcochlin-R as described previously [19]. To express mouse cochlin fused to myc-tag at N-terminus and human IgG-Fc at C-terminus (cochlin-Fc), mouse cochlin cDNAs were amplified by PCR and inserted between the EcoRI and XhoI sites of the pCAGGS-Fc vector as previously reported [44]. The cDNAs containing genes encoding cochlin (UniProtKB-Q62507, amino acid residues 27–552 for FL, residues 130–552 for ΔLCCL_a, residues 153–552 for ΔLCCL_b, residues 158–552 for ΔLCCL_c, residues 27–365 for ΔvWA2, residues 27–130 for LCCL_a, residues 27–153 for LCCL_b, residues 27–158 for LCCL_c, and residues 355–552 for vWA2) (Supplementary Figure S2) from *Mus musculus*, which lacks an N-terminal signal sequence, were fused to the myc-tag at the N-terminus, and a human IgG-Fc region at the C-terminus was inserted into the expression vector pCAGGS (Addgene, Watertown, MA, U.S.A.) and transfected into the CHO cells using the Lipofectamine 2000 reagent (Invitrogen, Carlsbad, CA, U.S.A.). The residues expressed for truncation mutants were determined based on the secondary structure predicted by PSIPRED (http://bioinf.cs.ucl.ac.uk/psipred/). The recombinant proteins were purified from the culture medium of transfected cells using a Monofinity A column (Genscript, Jiangsu, China), followed by concentration and buffer exchange into PBS using an ultrafiltration membrane (Amicon-Ultra 30K, Merck, Darmstadt, Germany). The cDNA encoding cochlin FL (residues 27–552) from *Mus musculus*, which was fused to a C-terminal PreScission protease (Cytiva, Marlborough, MA, U.S.A.) cleavage site located upstream of the protein A-tag, was inserted into the expression vector pMT/BiP/V5-His of the *Drosophila* expression system (Invitrogen) and transfected into *Drosophila* S2 cells. Protein expression was induced by the addition of 0.5 mM CuSO_4_, and the recombinant proteins were purified from the culture medium using IgG-Sepharose 6 FF (GE Healthcare, Marlborough, MA, U.S.A.), followed by concentration and buffer exchange into PBS using an ultrafiltration membrane. Experiments using human materials and genetic recombination experiments were conducted in accordance with a comprehensive, high quality care program, which has been approved by the Life Science Research Committee of the Graduate School of Frontier Sciences of The University of Tokyo guided by the Life Science Committee of The University of Tokyo.

### Indirect ELISA

Biotinylated GAGs were diluted to 40 ng/ml with 10 mM sodium phosphate, pH 7.4, containing 150 mM NaCl and 0.1% w/v Tween 20 (PBS-T), applied to streptavidin-coated 96-well plates (Thermo Fisher Scientific), and rinsed with PBS-T. Purified cochlin solutions expressed by CHO cells at 1.5 µg/ml in PBS-T were added to the wells and incubated at 4°C overnight. After washing with PBS-T, 100 µl horseradish peroxidase (HRP)-conjugated anti-human IgG-Fc antibody was added and incubated for 1 h at room temperature. After washing with PBS-T, 100 µl of ABTS (5120-0032, Sera Care, Milfold, MA, U.S.A.) was added to each well and incubated for 20 min at room temperature. Absorbance was measured at a wavelength of 405 nm. Each experiment was conducted in duplicate. In case of binding inhibitory assay with several GAGs, mCOCH(FL)-Fc was preincubated with the indicated concentration of an inhibitor for 2 h at 20°C and then measured the binding of mutated PNA-Fc (10 µg/ml) to immobilized heparin was performed as described above.

### Binding kinetics analysis

This experiment was conducted using Biacore X-100 (GE Healthcare). Biotinylated GAGs were immobilized on a sensor chip SA (GE Healthcare), according to the manufacturer's instructions. The binding experiments were conducted using GAG-immobilized sensor chips and various concentrations (1.25–10 µg/ml, two-fold serial dilution) of the purified cochlin solutions. Affinity parameters, *ka*, *kd*, and *K_D_* were determined using Biacore evaluation software (GE Healthcare) using a 1 : 1 binding model. The *K*_D_ value was calculated as *ka*/*kd*.

### Binding analysis with disaccharide GAG array

Sugar chips immobilized with heparin or synthetic sulfated disaccharides derived from HS, CS, and DS were purchased from SUDx-Biotec (Kagoshima, Japan) as previously described [25]. Briefly, synthetic disaccharides having a lipoyl group were used as ligands. Lipoic acid is reduced to dihydrolipoic acid, resulting in two SH groups in the molecule. SH groups of sugar derivatives were readily adsorbed on the surface of gold-coated tips. The sugar chip was set on a prism with refractive oil (nD = 1.518, Cargille Laboratories, Cedar Grove, NJ, U.S.A.) in an SPR apparatus SPR670M (Moritex, Saitama, Japan). SPR measurements were conducted at room temperature, according to the manufacturer's instructions, using various concentrations of purified cochlin solutions expressed by the S2 cells. The cochlin-containing solution was applied and washed with running buffer. The difference in the response between the equilibrium before running and after washing was recorded as the binding signal.

### Flow cytometry

Cells were grown to 70%–80% confluence in appropriate growth media and harvested in PBS containing 10 mM EDTA. Cells were incubated with ΔLCCL_a-Fc (10 µg/ml in PBS containing 0.5% w/v BSA and 0.1% w/v NaN_3_) for 30 min at 4°C, and binding was analyzed using a FACSCalibur (BD Biosciences, Franklin Lakes, NJ, U.S.A.) after secondary incubation with a PE-conjugated anti-human IgG-Fc antibody.

### Pull-down assay and lectin blotting

DU145 cells cultured confluently in three 10 cm dishes were harvested and re-suspended in lysis buffer [50 mM Tris–HCl (pH 7.5), 150 mM NaCl, 1 mM EDTA, 0.5% w/v Tween 20, 0.5% w/v Triton-X100, and 1/100 volume of protease inhibitor cocktail (539131, Merck)] and rotated for 30 min at 4°C. The whole-cell lysate was centrifuged at 15 000 rpm for 20 min, and the supernatant was collected in a new tube. Beit beads were prepared by mixing 40 µl of Protein G-Sepharose (GE Healthcare) and 10 µg of ΔLCCL_a-Fc in 500 µl of TBS-T (20 mM Tris–HCl, pH 7.5, containing 0.15 M NaCl and 0.1% w/v Tween 20) for 1 h at 4°C. The beads were washed thrice with TBS-T. The cell lysate and bait beads were mixed and incubated overnight at 4°C. The beads were washed twice with TBS-T. Forty microlitres of elution buffer (50 mM Tris–HCl, pH 7.5, 3 M NaCl) was added to the beads and the tube was centrifuged at 15 000 rpm for 5 min. The eluted solution was mixed with 8 µl of 6× SDS sample buffer, heated at 98°C for 7 min, and subjected to SDS–PAGE at 200 V for 60 min. The gels were subjected to silver staining or blotting onto a PVDF membrane blocked with Blocking-One (Nacalai Tesque, Kyoto, Japan). To detect the sugar ligand of cochlin, the membranes were incubated overnight with the cochlin mutant, ΔLCCL_a-Fc (80 ng/ml in Blocking-One) at 4°C. The membranes were then washed thrice with TBS-T and incubated with HRP-conjugated anti-human IgG-Fc antibodies (0.1 µg/ml in TBS-T) at room temperature for 3 h. For visualization, ECL prime (Amersham Bioscience, Buckinghamshire, U.K.) was used as a substrate for HRP, and chemiluminescence was detected using Image Quant LAS4000 (GE healthcare).

### Immunohistochemical staining of the GAG-modified cell lines and histochemical analysis

PgsA and PgsC cells were fixed on coverslips with 4% w/v paraformaldehyde for 40 min at room temperature. After washing with PBS, samples were blocked with PBS containing 3% w/v BSA for 60 min, followed by incubation with ΔLCCL_a-Fc (4 µg/ml in PBS) for 2 h at room temperature. The resulting coverslips were then incubated with DAPI and DyLight 550-conjugated anti-human IgG antibody (ab96908, Abcam, Cambridge, U.K.) for 1 h, and the coverslips were mounted on glass slides using Immu-Mount (Thermo Fisher Scientific). Images were taken using a BZ-X710 all-in-one fluorescence microscope (Keyence, Osaka, Japan). Cardiac specimens from autopsy cases were fixed in 10% w/v neutral buffered formalin and 3 µm thin sections were prepared after the paraffin-embedded process. The deparaffinized thin sections were treated with Target Retrieval Solution and High pH (Dako, Santa Clara, CA, U.S.A.), and multicolor immunostaining was conducted using the cochlin mutant ΔvWA2-Fc (0.1 µg/ml in PBS), antibodies to mast cell molecules (serglycin and mast cell tryptase), and eFluor 660 labeled anti-α-smooth muscle actin (SMA) antibody (1A4, Thermo Fisher Scientific). Mouse monoclonal anti-human mast cell tryptase antibody (AA1, Abcam) or polyclonal rabbit anti-human serglycin antibody (LS-B15450, LSBio, Seattle, WA, U.S.A.) was reacted overnight at 7°C, the binding of which was detected using an Alexa Fluo 488 Tyramide SuperBoost Kit (Thermo Fisher Scientific). After the stripping wash for primary antibodies, cochlin ΔvWA2-Fc (0.1 µg/ml in PBS) was incubated overnight at room temperature, and specific binding was detected through the reaction of rabbit anti-Myc-tag polyclonal antibody (562-5, MBL, Tokyo, Japan) and Alexa Fluor 555 plus anti-rabbit IgG antibody (A32732, Thermo Fisher Scientific). In addition, an anti-SMA antibody was used to detect the vascular smooth muscle walls. Coverslips were mounted on glass slides using Immu-Mount (Thermo Fisher Scientific). Informed consent has been obtained from all individuals included in this study and immunohistochemical staining for the autopsy samples obtained from this study was approved by the Ethics committee of the Graduate School of Medicine, Chiba University (Research subject number: 2218).

### Statistical analyses

The data of indirect ELISA, GAG array, binding kinetics analyses, and flow cytometry were expressed as means ± SE from three or more experiments.

## Data Availability

The data that support the findings of this study are available from the corresponding author, [K.Y.], upon reasonable request.

## Open Access

Open access for this article was enabled by the participation of University of Tokyo in an all-inclusive *Read & Publish* agreement with Portland Press and the Biochemical Society under a transformative agreement with Individual.

## Abbreviations

Ab: antibody
CHO: Chinese hamster ovary
CS: Chondroitin sulfate
CSS: Charcoal-stripped serum
DFNA9: Autosomal dominant nonsyndromic sensorineural deafness 9
DS: Dermatan sulfate
ECM: Extracellular matrix
ELISA: Enzyme-linked immunosorbent assay
FBS: Fetal bovine serum
Fc: human IgG-Fc
FGF: Fibroblast growth factor
FL: Full length
GAG: Glycosaminoglycan
GlcA: glucuronic acid
GlcN: Glucosamin
GlcNAc: N-acetylglucosamine
HA: Hyaluronic acid
HRP: Horseradish peroxidase
HS: Heparan sulfate
*ka*: Association rate constant
*kd*: Dissociation rate constant
*K_D_*: Equilibrium dissociation constant
KS: Keratan sulfate
LCCL: Limulus factor C, cochlin, and late gestation lung protein Lgl1
MS: Mass spectrometry
PBS: phosphate buffered saline
ProA: Protein A
PVDF: Polyvinylidene difluoride
SDS–PAGE: Sodium dodesylsulfate-polyacrylamide gel electrophoresis
SMA: Smooth muscle actin
SPR: Surface plasmon resonance
vWA: von Willebrand factor A
ΔS: desulfated
