# Supplementary material for: Construction of mouse cochlin mutants with different GAG-binding specificities and their use for immunohistochemistry
Source: Biochem J. 2023 Jan 6;480(1):41–56. doi: 10.1042/BCJ20220339 (PMC9987951; doi:10.1042/BCJ20220339)
Supplement: Supplementary Material [file BCJ-480-41-s1.pdf]

Supplementary Figure Legends

Supplementary Figure 1. Binding of the cochlin mutants to various glycans. Binding was examined by evanescent-field fluorescence detection using a microarray containing 16 monosaccharides, 59 oligosaccharides including six GAGs, HA, chondroitin sulfate A (CSA), dermatan sulfate (also called CSB), heparan sulfate (HS), heparin (HP), and keratan sulfate (KS), and 21 glycoproteins [24]. Sulfated oligosaccharides and sulfated glycosaminoglycans are highlighted in yellow. Mouse cochlin(FL)-Fc specifically bound to heparin and did not bind to any other spots. Values represent mean ± standard deviation (n = 2).

Supplementary Figure 2. Inhibition assay of cochlin-Fc binding to immobilized heparin by several GAGs. Mouse cochlin(FL)-Fc was preincubated with the indicated concentration of GAG for 1 h at 20 °C and then measured the binding of cochlin-Fc to immobilized heparin was performed. Values represent mean ± standard deviation (n = 3). DS, CSC, CSD showed like that of CSA.

Supplementary Figure 3. The expressed region of truncated mutants Eight truncation mutants of mouse cochlin were designed and prepared to investigate regions

that contribute to GAG recognition. The residues expressed for truncation mutants were determined based on the results of the secondary structure prediction by PSIPRED (http://bioinf.cs.ucl.ac.uk/psipred/). The amino acid sequence of basic amino acid-rich region between the LCCL and vWA1 domains (from K149 to K161) has been described.

Supplementary Figure 4. GAG-binding specificities of cochlin-Fc mutants. The binding activity of each cochlin mutant fused to human IgG-Fc (ΔLCCL_b; residues 153–552, LCCL_a; residues 27–130, LCCL_b; residues 27–153, LCCL_c residues 27–158 or vWA2; residues 355–552) was examined by indirect ELISA. Biotinylated GAGs were immobilized on streptavidin-coated microtiter plates and cochlin mutants were added to the wells at a concentration of 1.5 μg/mL. Values represent mean ± standard deviation (n = 2).

Supplementary Figure 5. Schematic illustration of the mechanism by which cochlin recognizes several GAGs. It was suggested that the vWA1 domain was essential for the binding of cochlin to GAGs, and the vWA2 domain and the loop region between the LCCL and vWA1 domains were involved in the interaction. The loop region specifically recognizes highly sulfated GAGs by cochlin (FL) in a coordinated manner.

GAG-binding specificity broadened when cochlin lacked the vWA2 domain (ΔvWA2). An LCCL-truncation mutant of cochlin (ΔLCCL_a) showed highly specific binding to heparin and CSE.

Supplementementary Figure 6. Electrostatic surface potential mapped on a homology model of vWA1 and vWA2 domains of mouse cochlin. The 3-dimensional homology model was predicted by SWISS-MODEL (https://swissmodel.expasy.org/interactive) using the structural information of PTMP1 (PDBID: 4CN9), which was predicted as the optimal model. The electrostatic surface potential, calculated using APBS (https://www.poissonboltzmann.org), was visualized using CueMol (http://www.cuemol.org). Positively and negatively charged surfaces are colored in blue and red, respectively.

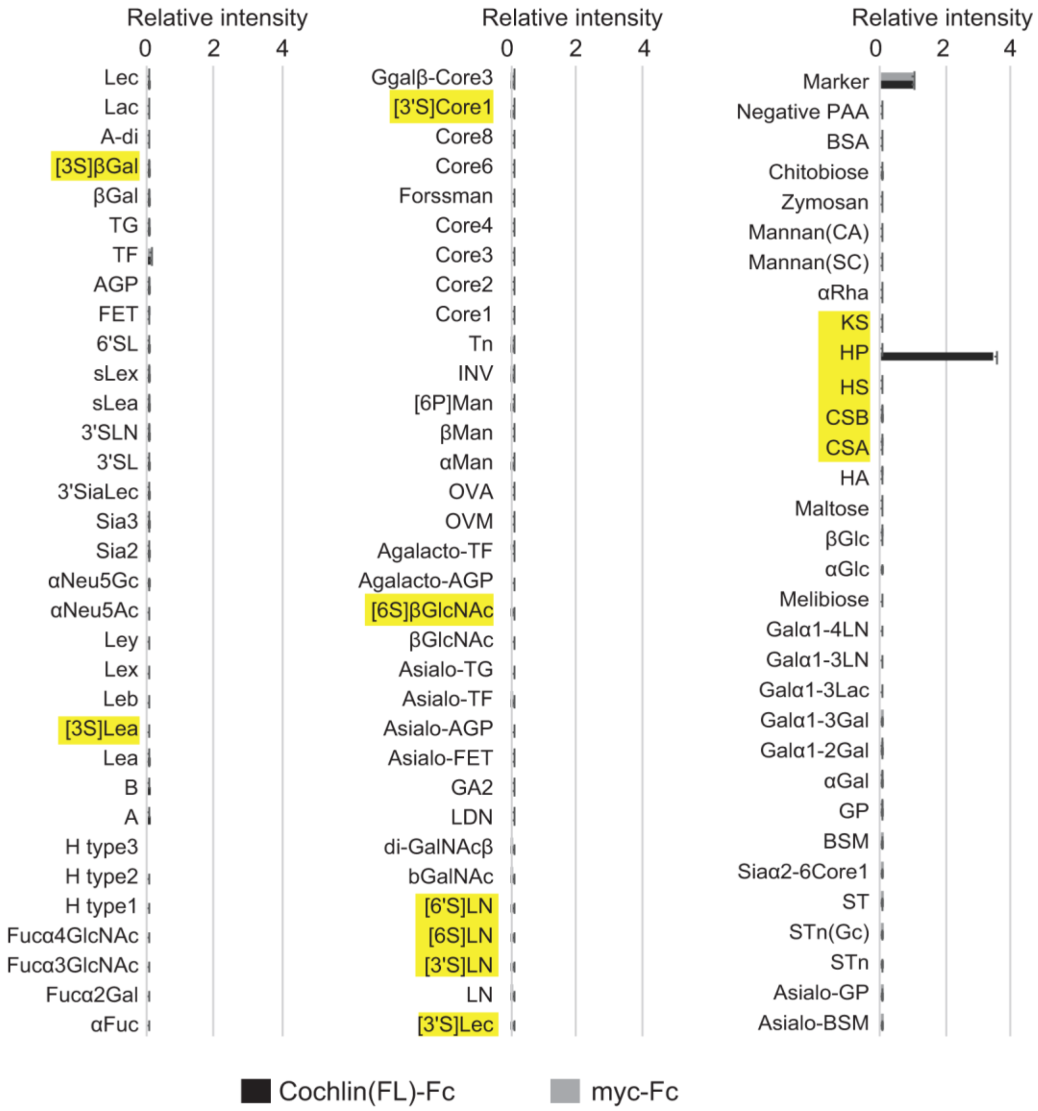

Relative intensity
0
2
4
Lec
Lac
A-di
[3S]βGal
βGal
TG
TF
AGP
FET
6'SL
sLex
sLea
3'SLN
3'SL
3'SiaLec
Sia3
Sia2
αNeu5Gc
αNeu5Ac
Ley
Lex
Leb
[3S]Lea
Lea
B
A
H type3
H type2
H type1
Fucα4GlcNAc
Fucα3GlcNAc
Fucα2Gal
αFuc
Relative intensity
0
2
4
Ggalβ-Core3
[3'S]Core1
Core8
Core6
Forssman
Core4
Core3
Core2
Core1
Tn
INV
[6P]Man
βMan
αMan
OVA
OVM
Agalacto-TF
Agalacto-AGP
[6S]βGlcNAc
βGlcNAc
Asialo-TG
Asialo-TF
Asialo-AGP
Asialo-FET
GA2
LDN
di-GalNAcβ
bGalNAc
[6'S]LN
[6S]LN
[3'S]LN
LN
[3'S]Lec
Relative intensity
0
2
4
Marker
Negative PAA
BSA
Chitobiose
Zymosan
Mannan(CA)
Mannan(SC)
αRha
KS
HP
HS
CSB
CSA
HA
Maltose
βGlc
αGlc
Melibiose
Galα1-4LN
Galα1-3LN
Galα1-3Lac
Galα1-3Gal
Galα1-2Gal
αGal
GP
BSM
Siaα2-6Core1
ST
STn(Gc)
STn
Asialo-GP
Asialo-BSM
Cochlin(FL)-Fc
myc-Fc

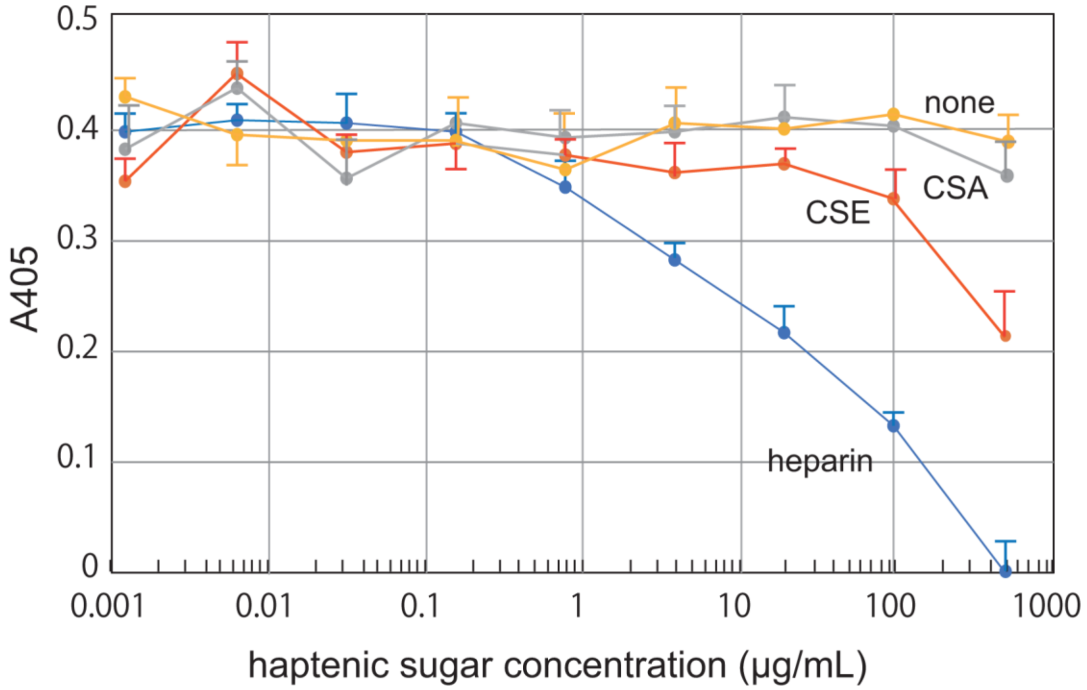
0.5
0.4
0.3
0.2
0.1
0
A405
0.001
0.01
0.1
1
10
100
1000
haptenic sugar concentration (μg/mL)
none
CSA
CSE
heparin

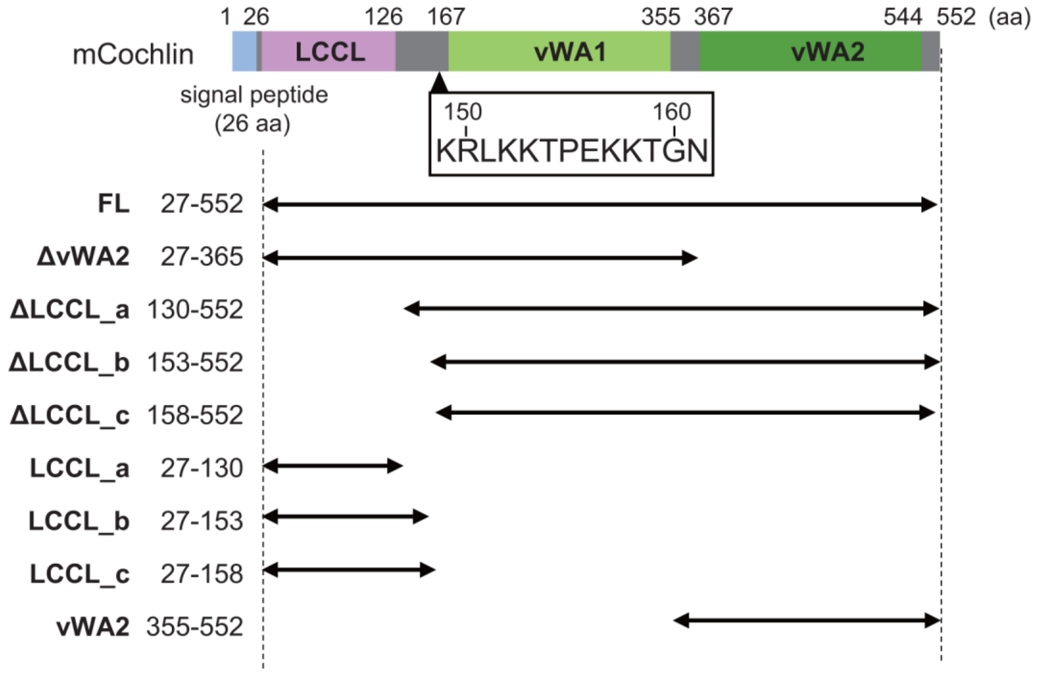

1
26
126
167
355
367
544
552
(aa)
mCochlin
LCCL
vWA1
vWA2
signal peptide
(26 aa)
150
160
KRLKKTPEKKTGN
FL 27-552
ΔvWA2 27-365
ΔLCCL_a 130-552
ΔLCCL_b 153-552
ΔLCCL_c 158-552
LCCL_a 27-130
LCCL_b 27-153
LCCL_c 27-158
vWA2 355-552

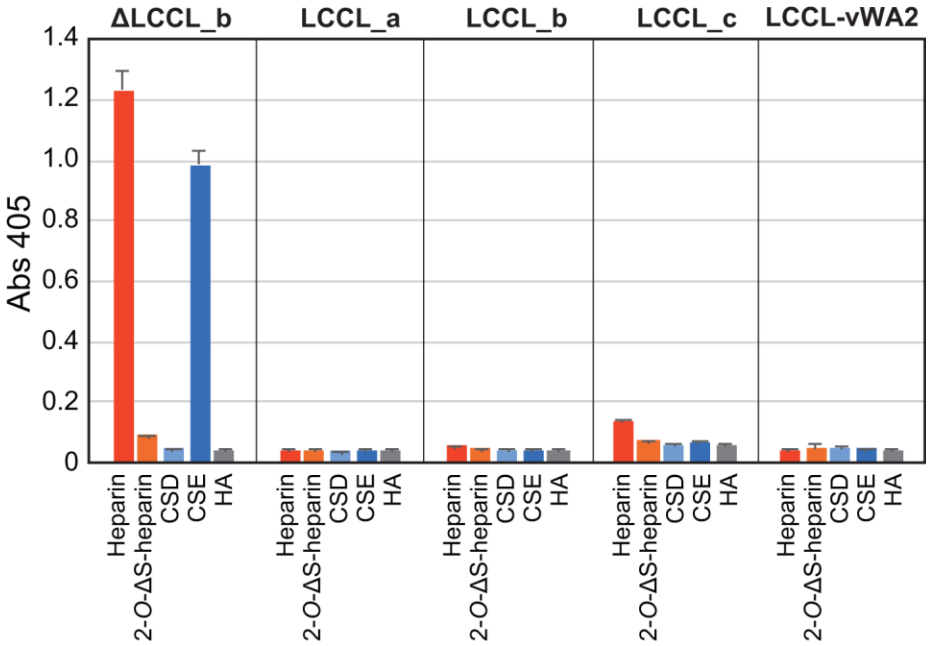

ΔLCCL_b
LCCL_a
LCCL_b
LCCL_c
LCCL-vWA2
Abs 405
1.4
1.2
1.0
0.8
0.6
0.4
0.2
0
Heparin
2-O-ΔS-heparin
CSD
CSE
HA

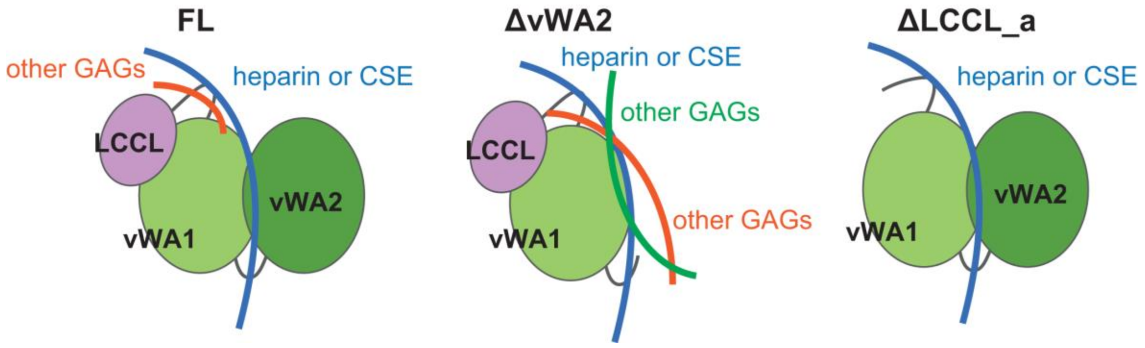
FL
other GAGs
heparin or CSE
LCCL
vWA1
vWA2
ΔvWA2
heparin or CSE
other GAGs
LCCL
vWA1
other GAGs
ΔLCCL_a
heparin or CSE
vWA1
vWA2

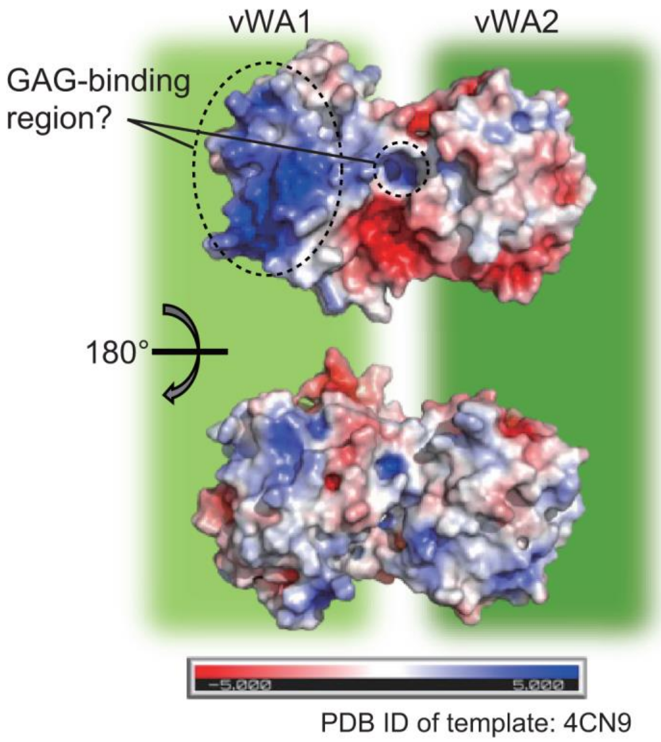
vWA1
vWA2
GAG-binding
region?
180°
-5.000
5.000
PDB ID of template: 4CN9
